# Invasive investigation: uptake and transport of l-leucine in the gill epithelium of crustaceans

**DOI:** 10.1093/conphys/coad015

**Published:** 2023-04-22

**Authors:** Robert A Griffin, Aaron Boyd, Alyssa Weinrauch, Tamzin A Blewett

**Affiliations:** Department of Biological Sciences, University of Alberta, 116 St & 85 Ave, Edmonton, AB, Canada T6G 2R3; Bamfield Marine Science Centre, 100 Pachena Rd, Bamfield BC, Canada V0R 1B0; Department of Biological Sciences, University of Alberta, 116 St & 85 Ave, Edmonton, AB, Canada T6G 2R3; Bamfield Marine Science Centre, 100 Pachena Rd, Bamfield BC, Canada V0R 1B0; Bamfield Marine Science Centre, 100 Pachena Rd, Bamfield BC, Canada V0R 1B0; Deptarment of Biological Sciences, University of Manitoba, 66 Chancellors Cir, Winnipeg, MB, Canada R3T 2N2; Department of Biological Sciences, University of Alberta, 116 St & 85 Ave, Edmonton, AB, Canada T6G 2R3; Bamfield Marine Science Centre, 100 Pachena Rd, Bamfield BC, Canada V0R 1B0

**Keywords:** invasive species nutrient uptake, branchial transport, Arthropod

## Abstract

Many aquatic species are well known as extremely successful invaders. The green crab (*Carcinus maenas)* is an arthropod native to European waters; however, it is now known to be a globally invasive species. Recently, it was discovered that the *C. maenas* could transport nutrients in the form of amino acids across their gill from the surrounding environment, a feat previously thought to be impossible in arthropods. We compared the ability for branchial amino acid transport of crustacean's native to Canadian Pacific waters to that of the invasive *C. maenas*, determining if this was a novel pathway in an extremely successful invasive species, or a shared trait among crustaceans. Active transport of l-leucine was exhibited in *C. maenas, Metacarcinus gracilis*, *Metacarcinus magister*, and *Cancer productus* across their gill epithelia. *Carcinus maenas* exhibited the highest maximum rate of branchial l-leucine transport at 53.7 ± 6.24 nmolg^−1^ h^−1^, over twice the rate of two native Canadian crustaceans. We also examined the influence of feeding, gill specificity, and organ accumulation of l-leucine. Feeding events displayed a heavy influence on the branchial transport rate of amino acids, increasing l-leucine transport rates by up to 10-fold in *C. maenas*. l-leucine displayed a significantly higher accumulation rate in the gills of *C. maenas* compared to the rest of the body at 4.15 ± 0.78 nmolg^−1^ h^−1^, with the stomach, hepatopancreas, eyestalks, muscle tissue, carapace and heart muscle exhibiting accumulation under 0.15 nmolg^−1^ h^−1^. For the first time, the novel transport of amino acids in Canadian native arthropods is described, suggesting that branchial amino acid transport is a shared trait among arthropods, contrary to existing literature. Further investigation is required to determine the influence of environmental temperature and salinity on transport in each species to outline any competitive advantages of the invasive *C. maenas* in a fluctuating estuarine environment.

## Teaser

Branchial amino acid uptake is evaluated in the invasive *Carcinus maenas* and compared to native Canadian crustaceans. l-leucine transport and accumulation throughout the body of the green crab is also evaluated in various gill and organ tissues in fed and fasted states.

## Introduction

Native to northeast Atlantic waters and the Baltic Sea, the European green crab (*Carcinus maenas*) is now found in coastal waters worldwide, including the Pacific and Atlantic coasts of Canada. Primary observations of the *C. maenas'* arrival on Pacific North American coasts were in 1989 in San Francisco Bay, CA, spreading 300 km north to Coos Bay, OR, by 1997, and quickly spreading to Vancouver Island, BC, by 1998 (Jamieson *et al.*, 1998). The expansion of this invasive population has since resulted in severe ecological impacts on Canadian native species and the surrounding ecosystem (Grosholz *et al.*, 2000; Matheson *et al.*, 2016). Despite their more recent introduction on the Canadian Pacific coast, juvenile *C. maenas* outcompete juvenile Dungeness crab (*Metacarcinus magister*) for habitat and food resulting in population decline and over $6 million in losses per year to the British Columbia decapod fishery (Colautti *et al.*, 2006). Comparatively, the well-established Atlantic population of *C. maenas* displaces the American lobster (*Homarus americanus*), resulting in a commercial loss of $44 to 114 million per year (Colautti *et al.*, 2006). Predation and hunting practices of *C. maenas* are also particularly destructive to the native ecosystem. Canadian bivalve populations experience reduced recruitment and survival rates due to significant predation, with bivalve populations exhibiting 5- to 10-fold decrease in the presence of *C. maenas* (Grosholz *et al.*, 2000; Tan and Beal, 2015; Ens *et al.*, 2022). While scavenging for bivalves, *C. maenas* also causes significant damage to Eelgrass (*Zostera* spp.) meadows that many estuarine organisms rely on for food and shelter. In Atlantic Canada, eelgrass meadows have experienced biomass declines between 50% and 100% when *C. maenas* invades their ecosystem (Ens *et al.*, 2022). In an attempt to preserve native species in the Canadian Pacific, researchers are often focused on slowing the growth of existing *C. maenas* populations to mitigate spread.

While some mitigation efforts have focused on the utilization of chemical agents, such as carbaryl and lindane to target invasive *C. maenas* populations, these agents are non-specific and inadvertently target native arthropods (Hanks, 1961; Iliff *et al.*, 2019; Ens *et al.*, 2022). As a result, current mitigation efforts largely rely on manual trapping, with focus on early detection and preemptive capture. Monitoring programs often attempt to pinpoint trapping areas of particular risk to *C. maenas* invasion, evaluating their habitat preference and physiological parameters to native organisms. In order to accurately predict the spread of *C. maenas*, it is vital to understand all factors that may influence the survival of both *C. maenas* and native organisms. However, when evaluating the interactions between native and invasive crustacean species, the role of waterborne amino acids on survival has been largely overlooked. Total amino acid concentrations generally range from 0.1 to 1 μM in the open ocean and increase to 2 μM or greater in estuaries, surrounding marine organisms in free dissolved nutrients (Stephens, 1968; Stephens, 1988). l-leucine in particular has been measured at approximately 8% of the total combined amino acid concentrations in the Pacific surface waters (Bada *et al.*, 1982). Notably, many marine invertebrates are capable of absorbing amino acids from the environment at concentrations as low as 50 nM, which can represent a significant source of their total nutrients (Wright, 1982; Preston, 1993). However, arthropods were historically excluded from this group due to the perceived impermeability of their exoskeleton (Preston, 1993; Chan *et al.*, 2019). Indeed, it was not until recently that the gills of arthropods were found to be able to extract free amino acids (i.e. l-leucine) from the aqueous environment similarly to other marine invertebrates (Blewett and Goss, 2017).

The gills are an ideal transport organ given their direct exposure to the environment, large surface area, and relatively thin membrane for ion transport, in conjunction with high rates of haemolymph perfusion (Henry *et al.*, 2012). The physiological roles of marine crustacean gills are separated into anterior (i.e. gills 2–5) and posterior gills (i.e. pairs 6–9), functioning primarily for respiration or ionoregulation, respectively (Towle *et al.*, 1997; Freire *et al.*, 2008). Research suggests that there are likely two transport pathways for l-leucine across the gill epithelium of *C. maenas* that are concentration-dependent and display both high and low affinity for this amino acid (Blewett and Goss, 2017). However, it is currently unknown if this ability extends to other species of crustaceans, how environmental stimuli can impact branchial transport, and the destination of transported amino acids within the crustacean body.

The green crab readily acclimates to a variety of environments, including fluctuating estuarine environments (Havel *et al.*, 2015), making it a globally successful invasive crustacean. These crabs can tightly regulate osmolytes and in fact, amino acids are known to play an important role in salinity tolerance and survival (Abe *et al.*, 1999). Thus, we sought to determine if the ability of *C. maenas* to transport nutrients across the gill epithelium was unique to this species and in turn a contributing factor to their survival and invasive success. To this end, we determined the extent to which other native crustaceans (e.g. *Metacarcinus gracilis* (graceful crab), *Cancer productus* (red rock crab), and *Metacarcinus magister* (Dungeness crab)), of the Pacific can transport amino acids, such as l-leucine across the gill epithelium in comparison to *C. maenas*. We hypothesized that arthropod species native to the Pacific coast of Canada would be able to transport l-leucine across the gill epithelium alongside *C. maenas* due to their shared gill structures, but with a lower capacity to do so, given *C. maenas*' increased ability to regulate internal osmolytes (Leignel *et al.*, 2014). To address this hypothesis, we characterized waterborne amino acid transport using gill perfusion techniques to elucidate the transport rate of l-leucine in four crustaceans' species, evaluated the influence of feeding events on branchial amino acid transport using both recently fed and fasted crustaceans, and performed whole body bioaccumulation exposures in *C. maenas* to evaluate waterborne amino acid transport and dispersion throughout crustacean gills and organs.

## Materials and Methods

### Animal collection

Invasive adult male *C. maenas* weighing on average 101 ± 16.5 g and adult male native crab species (*M. gracilis* 174 ± 41.4 g, *C. productus* 453 ± 156 g, and *M. magister* 528 ± 116 g) were collected in accordance with Fisheries and Oceans Canada license XR 1352020, off the western side of Vancouver Island in Barkley Sound (N49802.274–W125820.710 and N49801.749–W125821.515). Captured animals were transported to the Bamfield Marine Science Centre (Bamfield, BC). Animals were held in various mesh-covered outdoor 2000 L tanks, on a flow through system of filtered seawater pumped from the ocean (10.6 ± 0.4°C, 31.5 ± 1.3 ppt). Animals were subjected to natural summer lighting (~15 h light, ~ 9 h dark) and fed salmon to satiety every 3 days. A minimum of 7 days prior to experimentation, animals were transported into indoor carbon filtered tanks, fasted, and held at 31.7 ± 0.2 ppt at 17.7 ± 0.8°C. All collection and holding was performed in accordance with University of Alberta, Bamfield Marine Sciences Centre, and Canadian Council on Animal Care guidelines.

### Gill perfusions

Gill perfusion experiments were performed in accordance with the methods described by Siebers *et al.* (1985) and Blewett and Goss (2017). Briefly, crabs were euthanized via a single spike to the ventral ganglion following 15 minutes on ice, allowing for anesthetization. Posterior gills (7–9), or anterior gill 5 were dissected for perfusion; however, only one gill per individual was used per treatment to avoid pseudo-replication. A range of PE tubing 80 to 210 (Intramedic, Clay Adams) was used based upon the gill size of each crab, and inserted into the afferent and efferent vessels of the gill, and the basal end of the gill was tightly clipped, creating a closed system. In addition to previous work with inulin perfusions (Blewett and Goss, 2017), red dye was perfused through preliminary test gills to ensure no leaks were found within gill clamps, indicating a closed system was being achieved prior to experimental perfusions. Gill perfusion was performed using an artificial hemolymph at 100% SW (saltwater) osmolarity (Table 1), (composition in mM: 470 NaCl, 12 CaCl_2_, 12 MgCl_2_, 11 KCl, 9 NaHCO_3_, 0.1 NH_4_Cl, 0.3 glucose, 0.1 glutathione, 0.5 glutamine, mOsm = 954 ± 2.6) at a rate of approximately 127 ± 1.7 μL/min using a 12-channel peristaltic pump (Fisherbrand FH100M; Fisher Scientific, USA). Gills were then placed in 50 mL of bathing solution comprised of seawater (32 ± 0.2 ppt, 17.7 ± 0.8°C) spiked with ^14^C radiolabelled l-leucine (Perkin Elmer, Boston, MA), at concentrations of 0, 1, 10, 100, and 1000 μM and perfused for 1 h, based on previous work by Blewett and Goss (2017). Each treatment was replicated between three and seven times, depending on individual species gill availability.

**Table 1:** Crab haemolymph, artificial perfusate and saltwater osmolarity for C. maenas, M. gracilis, M. magister, and C. productus. Values are displayed as averages ± SEM, in mmol/kg (n = 5)

	Osmolarity (mmol/kg)
Seawater (32 ppt)	971 ± 1.63
Artificial perfusate	954 ± 2.59
*C. maenas*	971 ± 12.4
*M. gracilis*	985 ± 16.1
*M. magister*	981 ± 13.8
*C. productus*	950 ± 18.1

In order to compare the influence of feeding on the branchial uptake of l-leucine, *C. maenas*, *M. gracilis* and *M. magister*, were fed salmon tissue to satiety in the hour leading up to euthanization. Posterior gills were then dissected and perfused in 2 μM radiolabelled l-leucine following the procedure above. The influence of feeding on the branchial uptake of l-leucine was not evaluated in *C. productus* as a result of limited animal availability. Each treatment was replicated between four and six times, depending on individual species gill availability.

A 1-mL subsample of perfusate was collected at the end of each treatment for radioisotope analysis and mixed with 4 mL of Optiphase (Perkin Elmer, MA) liquid scintillant. Individual beta emissions were measured via counts per minute (CPM) of each sample using a TriCarb 29 000 TR liquid Scintillation Analyzer. Gills were weighed and amino acid transport rates were then calculated for each gill (see *Calculations and Data Analysis*). All “zeros” were considered true zeros as gills were perfused without radioisotope and individual beta emissions were recorded at background levels.

**Figure 1:**
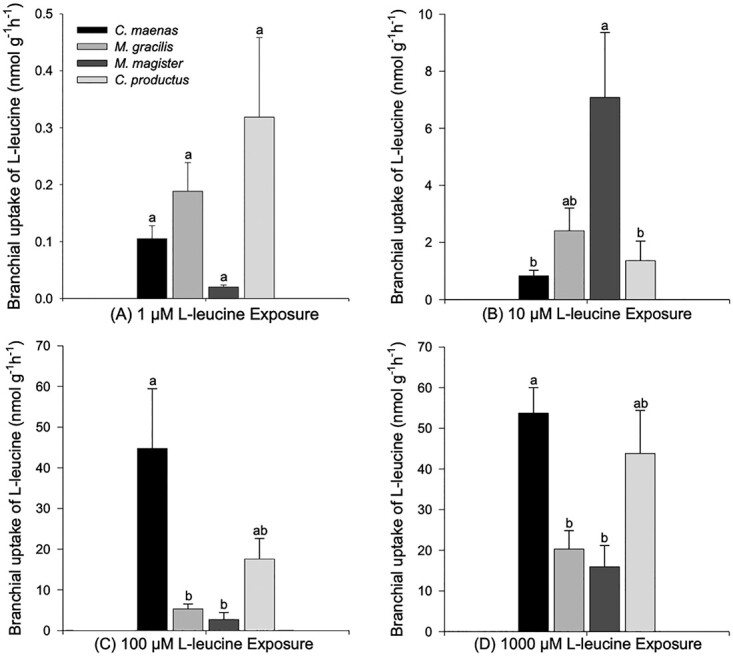
l-leucine transport in the posterior gills (7–9) of *Carcinus maenas*, *Metacarcinus gracilis*, *Metacarcinus magister*, and *Cancer productus* at waterborne l-leucine concentrations of (A) 1 μM, (B) 10 μM, (C) 100 μM, and (D) 1000 μM determined via gill perfusion. Values represent mean ± s.e.m (n = 4–7). A one-way ANOVA was conducted to compare the branchial uptake rate (nmol g-1 h-1) of species at each environmental concentration. p ≤ 0.05 was considered significant. Bars within concentration groups that share lettering are not considered statistically different, while bars that do not share lettering are considered statistically significant.

### Whole-body exposures

l-leucine was made from a concentrated l-leucine stock of 10 mM (Sigma Aldrich), and added to the seawater 24 h before experimentation to equilibrate. Thirty minutes before the start of the experiment, 1 μCi^−1^ of ^14^C radiolabelled l-leucine (Perkin Elmer, Boston, MA) was added to each exposure chamber for a final concentration in the chambers of 300 μM l-leucine. Water samples of 1 mL were taken at t = 0 and t = 24 to monitor radioisotope concentration. *Carcinus maenas* were starved for 4 days prior to exposure to whole animal experiments. Five individual crabs were then randomly placed into the containers and allowed to rest for 24 h in the aerated mixture. Upon termination of the exposure, crabs were washed (1 min) in a highly concentrated nonradiolabelled l-leucine wash (10 mM) for displacement of any radiolabelled leucine that was loosely bound to the outside of the animal, they were then washed in 100% seawater for 1 min, and immediately following that, crabs were placed on ice for 15 mins and euthanized as stated above. Once crabs were euthanized, they were dissected and the following organs were weighed and digested as described below for radioisotope analysis: gill 5 (G5), gill 8 (G8), stomach (ST), hepatopancreas (HP), eyestalk (ES), muscle (MU), carapace (CA) and haemolymph (HL).

In order to compare the impacts of feeding on l-leucine accumulation in the gills of *C. maenas*, the procedure above was also conducted using *C. maenas* that had been fed to satiety 1 h prior to experimental exposure. Five individual fasted crabs and five individual fed crabs were then randomly placed into the containers and allowed to rest for 24 h in the aerated mixture. Once crabs were euthanized, they were dissected and gills 2 to 9 (G2–G9) were weighed and digested as described below for radioisotope analysis.

### Tissue and water analysis

Tissues were weighed and collected in 20 mL scintillation vials and digested with 2N HNO_3_ (trace metal grade nitric acid, Sigma Aldrich) at the three to five times (exact volume recorded) the weight of the tissue. All vials were sealed and placed in an incubator at 65°C for 48 h, with vortexing after 24 h. Scintillation fluor was then added to the scintillation vials (Ultima Gold, Perkin Elmer, Waltham, MA) in a 5:1 ratio of volume to tissue and assayed for radioactive beta-emission counts on a TriCarb 29000 TR liquid Scintillation Analyzer. Samples were standardized to a common counting efficiency (quench corrected), using a quench curve constructed from tissue digests. Water samples (1 mL) were treated as above, where Optiphase (Perkin Elmer, MA) liquid fluor was added at a volume of 2:1 ratio of volume to water and run for emission counts on the Liquid Scintillation Analyzer above.

**Figure 2:**
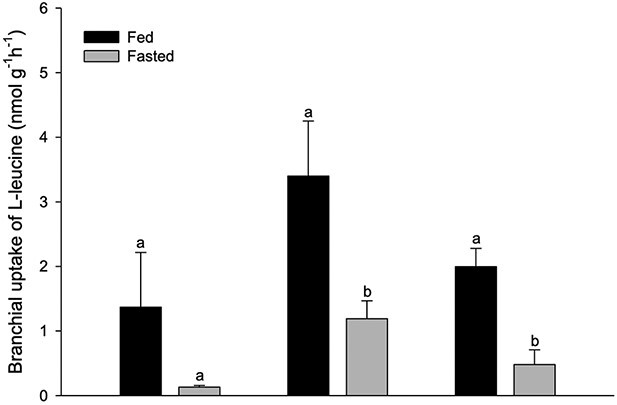
Fed vs Fasted branchial uptake of 2 μM l-leucine in the posterior gills (7–9) of *Carcinus maenas, Metacarcinus gracilis*, and *Metacarcinus magister*. Bar values represent mean ± s.e.m (n = 4–5). T-tests were conducted to compare the branchial uptake rate (nmol g-1 h-1) of each species in their fed and fasted states. p ≤ 0.05 was considered significant. Bars that do not share lettering display statistically significant fed and fasted values. Bars without lettering are considered to show no significant differences between fed and fasted uptake.

### Calculations and data analysis

Specific activity is the ratio of measured radioactivity of the stock solution to the total concentration (μM) of amino acid present in the experimental exposure:

\begin{align*} \mathrm{Specific}\ \mathrm{activity}=\frac{\mathrm{Stock}\ \mathrm{CPM}}{\mathrm{Stock}\ \mathrm{amino}\ \mathrm{acid}\ \mathrm{concentration}} \end{align*}

To calculate amino acid uptake, measured perfusate or tissue radioactivity (CPM) was divided by the specific activity (SA) of the exposure solution. To account for the variable gill sizes among species, amino acid concentration was converted into nmol g^−1^ based on individual gill weights. Finally, amino acid uptake (nmol g^−1^ h^−1^) or tissue accumulation (nmol g^−1^ h^−1^) was determined by accounting for total perfusion time in hours (t):

$$ \mathrm{amino}\ \mathrm{acid}\ \mathrm{uptake}=\frac{CPM}{SA}\times \frac{1}{g}\times \frac{1}{t} $$

Outliers were identified and removed using Grubbs' test, and l-leucine and concentration-dependent uptake was evaluated and plotted using SigmaPlot Version 11.0 (Systat Software, San Jose, CA). Uptake at each given amino acid concentration (μM) versus branchial transport rate (nmol g^−1^ h^−1^) for the four arthropod species at high and low environment concentrations were plotted on raw data using Sigmaplot Version 11.0.

**Figure 3:**
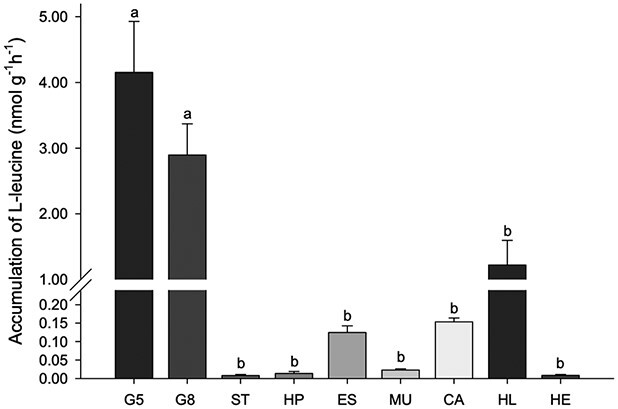
Organ accumulation of 2 μM l-leucine in the anterior gill 5 (G5), posterior gill 8 (G8), stomach (ST), hepatopancreas (HP), eyestalk (ES), muscle (MU), carapace (CA), haemolymph (HL), and heart (HE) of *Carcinus maenas*. Values represent mean ± s.e.m (n = 4–5). A one-way ANOVA was conducted to compare the accumulation of l-leucine (nmol g-1 h-1) within each specific organ. p ≤ 0.05 was considered significant. Bars that do not share lettering display statistically significant l-leucine accumulation values. Bars that share lettering are not considered statistically different.

**Figure 4:**
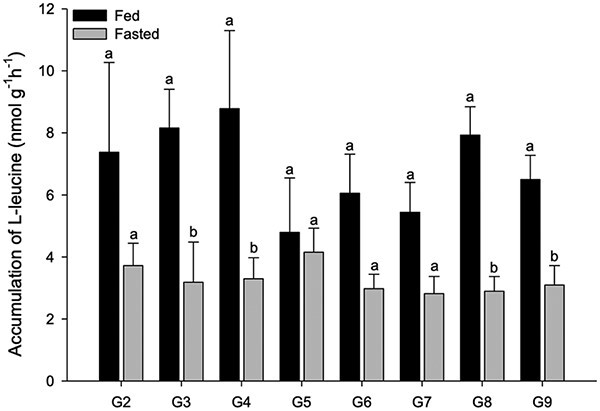
Gill accumulation of 2 μM l-leucine in the anterior gills (G2-G5) and posterior gills (G6-G9) of *Carcinus maenas* in both fasted and fed states. Values represent mean ± s.e.m (n = 4–5). A two-way ANOVA was conducted to compare the accumulation of l-leucine (nmol g-1 h-1) between gill number, and the influence of fed state. p ≤ 0.05 was considered significant. Data was transformed (Log10) in Sigmaplot 11.0 to achieve normality. No significant differences were seen in relation to gill number and l-leucine accumulation. Bars that do not share lettering display statistically significant l-leucine accumulation values between fed and fasted states. Bars without lettering are not considered statistically different between fed and fasted states.

All statistical analyses were performed in Sigmaplot Version 11.0. ANOVA and Holm-Sidak post hoc tests were conducted to identify any significant differences in transport rate between species at four environmental concentrations (Figure 1), and to evaluate differences in 24-h l-leucine accumulation in various *C. maenas* organs (Figure 3). Normality was ensured and *t* tests were performed to evaluate differences between fed and fasted l-leucine transport rates in each species (Figure 2). Whole body feeding state and gill number data were transformed in Sigmaplot 11.0. using the log transform function to achieve normality, a two-way ANOVA was then conducted to evaluate the influence of feeding, gill number, and the interaction between feeding and gill number on 24-h gill accumulation of l-leucine in *C. maenas* (Figure 4).

## Results

*Metacarcinus magister*, *C. maenas, C. productus* and *M. gracilis* exhibited branchial amino acid uptake in environmental concentrations ranging from 0 to 1000 μM. The highest branchial transport rate of l-leucine (Figure 1) was noted in *C. maenas* at the 1000-μM exposure (53.7 ± 6.24 nmol$\cdot $g^−1^$\cdot $h^−1^), exhibiting a significantly higher (p = 0.007, df = 15, f = 6.70) branchial transport rate when compared to *M. gracilis* (20.3 ± 4.53 nmolg^−1^ h^−1^) and *M. magister* (15.9 ± 5.22 nmol$\cdot $g^−1^$\cdot $h^−1^). While *C. maenas* showed no significant difference to *C. productus* (43.8 ± 10.6 nmol$\cdot $g^−1^$\cdot $h^−1^) at 1000 μM*.* Notably, no significant differences (p = 0.076) were seen between any crustacean’s branchial transport at 1 μM exposure. Contrarily, at 10 μM exposure, *M. magister* (7.08 ± 2.27 nmol$\cdot $g^−1^$\cdot $h^−1^) was noted to have significantly higher branchial transport of l-leucine (p = 0.014, df = 16, f = 5.25) compared to both *C. maenas* (0.83 ± 0.20 nmol$\cdot $g^−1^$\cdot $h^−1^) *C. productus* (1.36 ± 0.68 nmol$\cdot $g^−1^$\cdot $h^−1^). Finally, at 100 μM exposure *C. maenas* (44.8 ± 14.6 nmol$\cdot $g^−1^$\cdot $h^−1^) exhibited significantly higher branchial transport (one-way ANOVA, p = 0.03, df = 19, f = 3.74), when compared to both *M. gracilis* (5.31 ± 1.20 nmol$\cdot $g^−1^$\cdot $h^−1^) and *M. magister* (2.69 ± 1.73 nmolg^−1^ h^−1^).

*Metacarcinus gracilis* (p = 0.03, df = 7, t = 2.72) and *M. magister* (p = 0.005, df = 7, t = 4.00) exhibited significantly higher branchial uptake of l-leucine immediately after feeding, increasing from a fasted value of 1.19 ± 0.278 nmol$\cdot $g^−1^$\cdot $h^−1^ to 3.40 ± 0.853 nmol$\cdot $g^−1^$\cdot $h^−1^, and 0.48 ± 0.23 nmol$\cdot $g^−1^$\cdot $h^−1^ to 2.00 ± 0.28 nmol$\cdot $g^−1^$\cdot $h^−1^, respectively (Figure 2). While *C. maenas* did not exhibit a significant difference (p = 0.054) between fed and fasted gills, a general trend can be seen increasing an order of magnitude from fasted gills with a transport rate of 0.132 ± 0.025 nmol$\cdot $g^−1^$\cdot $h^−1^ to fed gills with a transport rate of 1.37 ± 0.85 nmol$\cdot $g^−1^$\cdot $h^−1^.

Notably, throughout *C. maenas* whole-body exposures gill 5 and gill 8 showed significantly higher (p < 0.001, df = 43, f = 19.5) uptake of l-leucine when compared with all other organs, with total accumulation rate at 4.15 ± 0.78 nmol$\cdot $g^−1^$\cdot $h^−1^ and 2.90 ± 0.47 nmol$\cdot $g^−1^$\cdot $h^−1^, respectively (Figure 3). No other significant differences (p > 0.05) are noted between the stomach (7.92 × 10^−3^ ± 2.86$\times$10^−3^ nmolg^−1^ h^−1^), hepatopancreas (1.34 × 10^−2^ ± 2.86 × 10^−3^ nmol$\cdot $g^−1^$\cdot $h^−1^), eyestalks (1.25 × 10^−1^ ± 1.81 × 10^−2^ nmol$\cdot $g^−1^$\cdot $h^−1^), muscle (2.27 × 10^−2^ ± 3.40 × 10^−3^ nmol$\cdot $g^−1^$\cdot $h^−1^), carapace (1.54 × 10^−1^ ± 1.04 × 10^−2^ nmol$\cdot $g^−1^$\cdot $h^−1^), haemolymph (1.22 ± 0.38 nmol$\cdot $g^−1^$\cdot $h^−1^) and heart (8.42 × 10^−3^ ± 2.39 × 10^−3^ nmol$\cdot $g^−1^$\cdot $h^−1^).

When evaluating how feeding state and gill number influence branchial l-leucine accumulation in *C. maenas*, no significant interaction was found between the fed state and gill number (p = 0.4). Likewise, no significant differences were seen between gill number and l-leucine accumulation in either fed or fasted gills (p > 0.05).

No significant differences were found between artificial perfusate, *C. maenas* haemolymph, *M. gracilis* haemolymph, *C. productus* haemolymph and *M. magister* haemolymph, or saltwater (32 ppt) osmolarity (Table 1).

## Discussion

Marine invertebrates utilize waterborne amino acids in various physiological purposes, such as osmoregulation and nutrient uptake, increasing their survival and optimal habitat range (Wright, 1982). Of the four arthropod species tested, all exhibited the ability to not only acquire amino acids over their gills, but to do so at rates akin to that of well-studied filter feeders (Stephens and Schinske, 1961; Rice *et al.*, 1980). This study compared the ability of three native Pacific arthropod species, *Metacarcinus gracilis*, *Cancer productus*, and *Metacarcinus magister,* to transport l-leucine across their gill epithelia, to that of the invasive green crab (*Carcinus maenas*). It was hypothesized that, given *C. maenas*' ability to acquire l-leucine from the environment, native Pacific arthropods would also share this ability and transport l-leucine across their gill epithelium but at a lower rate of transport than *C. maenas*. Our hypothesis was partially supported in that l-leucine was transported across both the anterior and posterior gill membranes of all four species. The noted uptake suggests that *M. gracilis*, *C. productus*, *M. magister* and *C. maenas* have the ability to actively transport l-leucine from the environment through their gills. However, *C. maenas* was only noted to have an increased capacity for transport over *M. magister* at high environmental concentrations of 100 and 1000 μM, showing a lower transport rate than *M. magister* at 10 μM and was otherwise comparable to the Pacific native species. Notably, after being transported from the environment, amino acids were shown to remain largely in the gill, with lower concentrations noted in the haemolymph and throughout the organs. Branchial amino acid transport rates were also shown to increase in *C. maenas*, *M. gracilis* and *M. magister* immediately following a feeding event.

### Branchial transport among species

Epithelial nutrient uptake in arthropods was previously thought to be an impossible process due to their hard and relatively impermeable exoskeleton (Preston, 1993; Chan *et al.*, 2019). However, Blewett and Goss (2017) provided evidence for branchial uptake of l-leucine in the invasive green crab at environmentally relevant amino acid concentrations. Notably, our results show that branchial amino acid uptake is not only displayed in a single arthropod species, but likely a shared trait among all marine crustaceans. *M. gracilis*, *C. productus*, *M. magister*, as well as *C. maenas* all display an active transport pathway for the uptake of free l-leucine environmental concentrations ranging from 0 to 1000 μM (Figure 1).

It was hypothesized that *C. maenas* would exhibit a higher capacity for transport than native species given the green crabs’ increased ability to regulate internal osmolarity; however, we did not observe this. At high concentrations of l-leucine (100 μM–1000 μM), *C. maenas* exhibited higher rates of transport compared to two of the three native species (i.e. *M. gracilis* and *M. magister*) (Figure 1). Interestingly, the invasive *C. maenas* shows significantly lower branchial amino acid transport when compared to *M. magister* at environmental concentrations of 10 μM. This suggests that the branchial uptake of l-leucine from the environment in *C. maenas* offers little competitive advantage over Canadian native crustaceans at ambient environmental amino acid concentrations. It was also previously noted that a low concentration (0–10 μM) pathway could offer greater physiological significance to arthropods based on normal environmental amino acid concentrations than the higher 100 to 1000 μM treatments (Blewett and Goss, 2017). Given that average estuarine amino acid concentrations are generally in the 2 μM range, a significantly higher rate of amino acid transport in *C. maenas* at environmental concentrations approaching 100 μM would rarely be utilized under normal environmental conditions (Stephens, 1968; Stephens, 1988). This further suggests that the invasive green crab would likely have no competitive advantage with respect to the branchial uptake rate of waterborne l-leucine when compared to the native Pacific species at common environmental amino acid concentrations. However, in isolated environmental instances that result in amino acids at concentrations approaching 100 μM and above (see below), *C. maenas* may have a competitive advantage over *M. gracilis* and *M. magister.*

### Feeding events and branchial transport

Throughout this study, we also sought to examine the influence of feeding events on branchial uptake of waterborne amino acids in order to understand the nutritional role of branchial amino acid uptake. Often, increased transport rates are indicative of an increased expression of transporters after an external stimulant. Interestingly, *M. gracilis* and *M. magister* both exhibited significant increases in branchial l-leucine uptake immediately after a feeding event (Figure 2). Similarly, even in the absence in statistical significance, a nearly 10-fold increase in branchial uptake can also be observed in *C. maenas* immediately after a feeding event (Figure 2). This suggests that the branchial uptake of waterborne amino acids in crustaceans is heavily influenced by a feeding event, displaying uptake rates ranging from nearly 3- to 10-fold that of fasted uptake. Comparatively, in perhaps the most notorious example of postprandial regulation of nutrient transport, the Burmese python shows a 5.7-fold increase in l-leucine uptake to maximize nutrient acquisition during times of feeding, and conserve digestive energy while fasting (Cox and Secor, 2008). In many vertebrates, including the Burmese python (Secor *et al.*, 2001), cholecystokinin/gastric-like peptides (CCK/gastric) increase after ingestion and act as regulatory peptides in the gastrointestinal endocrine system, stimulating pancreatic enzyme secretions (Larson and Vigna, 1983). Similarly, CCK/gastric peptides are found within the gastrointestinal system of *C. maenas* and other crustaceans, and in turn are shown to stimulate the release of digestive enzymes by the hepatopancreas in the freshwater crustacean *O. limosus* (Larson and Vigna, 1983; Resch-Sedlmeier and Sedlmeier, 1999). While the exact endocrine response pathway remains unknown, we propose that upon ingestion, the release of CCK/gastric peptides or other similar hormones into the haemolymph not only stimulates the release of digestive enzymes in the hepatopancreas but also induces the increased expression of transport proteins on the gill epithelia. The authors also suggest that during a feeding event, the creation of a localized “feeding cloud” could lead to a significantly higher environmental (>100 μM) concentration of waterborne amino acids, leeching out of the given food source and creating a microclimate in which upregulating branchial nutrient uptake could be extremely advantageous. Notably, postprandial oxygen consumption in crustaceans increases between 1.5- and 3-fold compared to resting levels, resulting in a high rate of energy production and consumption, increasing nutrient transport throughout the body (McGaw and Curtis, 2013). In conjunction with the creation of a feeding cloud, an increase in branchial amino acid transport rate would allow for the increased uptake of nutrients that would otherwise be lost to the surrounding environment; however, further research is required to confirm this theory.

### Uptake specificity and accumulation

While waterborne amino acid uptake has been studied in 10 different marine invertebrate taxa, now including four arthropod species, the identification of amino acid transporters in marine invertebrates relies heavily on mammalian models (Stephens, 1988; Glover *et al.*, 2011; Katayama *et al.*, 2016; Blewett and Goss, 2017). As such, little is known about the exact transporters present on invertebrate gill epithelia; however, most mechanistic evidence suggests a combination of SLC6 family Na^+^/AA cotransporters on the basolateral and apical membrane, allowing amino acid transport across the gill epithelia (Wright, 1987; Applebaum *et al.*, 2013; Blewett and Goss, 2017). It has been widely described that the epithelia of the posterior (i.e. 6–9) and anterior gills (i.e. 2–5) of euryhaline crabs are specialized for unique primary purposes (Towle and Weihrauch, 2001; Freire *et al.*, 2008; Blewett and Goss, 2017). While the anterior gills are believed to serve a mainly respiratory function owing to their relatively thin epithelia, the posterior gills are characterized by a thicker, mitochondrially rich epithelium (Freire *et al.*, 2008). The increased expression of mitochondria provides energy in the form of ATP to power the Na^+^/K^+^ATPase, driving an increased electrochemical gradient in the cell, thus creating the Na^+^/AA co-transport capacity of the posterior gills (Towle and Weihrauch, 2001). However, contrary to existing literature (Blewett and Goss, 2017), no significant differences were exhibited between the anterior and posterior transport of l-leucine in *C. maenas* (Figure 3), suggesting that regardless of the noted physiological differences, amino acid transport pathways appear uniform across all gills. This lack of gill specificity across physiologically different tissues could be explained in part by the presence of multiple unique amino acid pathways across epithelial surfaces, similar to marine echinoderms which exhibit 11 specific amino acid SLC6 family cotransporters throughout their epithelium and tube feet regardless of tissue specificity (Applebaum *et al.*, 2013). It is, therefore, challenging to draw a conclusive statement regarding the specificity of anterior and posterior *C. maenas* gills and their role in the transport of waterborne amino acids without further investigation.

Understanding the ability of individual gills to transport amino acids from the environment into the epithelia cells offers valuable information regarding the accumulation of waterborne amino acids in the body of marine crustaceans. However, gill transport rates offer little evidence regarding the dispersion and utilization of amino acids throughout the crustacean body. The tissue-specific distribution of transported l-leucine throughout the body of *C. maenas* was therefore also evaluated to understand the whole-body distribution of accumulated amino acids. Generally, marine organisms maintain their internal amino acid concentration at 10^3^ to 10^6^ fold higher than their external environment (Clark, 1968; Stevens and Preston, 1980). As suggested above, amino acids are transported across the gill epithelia into the haemolymph via Na^+^/AA cotransporters on the apical and basolateral membrane, before being transported throughout the body via the circulatory system. Therefore, as expected, waterborne amino acids were transported through gills 5 and 8 into the haemolymph of *C. maenas*, and further dispersed into the stomach, hepatopancreas, eyestalk, muscle tissue, carapace, and heart of *C. maenas*. While both gills 5 and 8 exhibit significantly higher accumulation of l-leucine compared to all other organs and the haemolymph, this could be due in part to l-leucine that was actively being acquired and sequestered in the epithelial cells at the end of the exposure period. Comparably, Kucukgulmez and Celik (2008) found no distinct differences in l-leucine accumulation between various muscle tissues and the hepatopancreas of the blue crab (*C. sapidus)*, indicating little specificity between l-leucine accumulation and unique organ tissues similar to the results of this study. After the gills, the highest rate of accumulation throughout the body can be seen in the haemolymph. This elevated level of free amino acids within the circulatory system of *C. maenas* after a 24-h exposure period suggests that the primary utilization of l-leucine within the green crab, may not be protein synthesis. In *C. maenas*, protein synthesis is known to return to fasted levels within 16 h of a single meal, peaking at a rate nearly four times that of fasted crustaceans (Carter and Mente, 2014). Carter and Mente (2014) also note no difference in protein synthesis rates in the gill, heart, leg, and claw of unfed and continually fed *C. maenas*. Thus, the metabolic fate of branchial acquired amino acids may largely depend on feeding regimes, suggesting that after a feeding event and increase in metabolic rate, l-leucine could serve as secondary nutrient source for protein synthesis. However, given the large retention of l-leucine in the gills and haemolymph in periods of fasting, in combination with research suggesting low protein conversion efficiency, and a maximum of 40% amino acids utilization for protein synthesis (Mente *et al.*, 2010), we suggest a primarily osmoregulatory function of branchial amino acid uptake.

It is therefore possible that these amino acids are being utilized as an osmolyte throughout the body, not unlike the osmoregulatory strategy of the Japanese mitten crab (*Eriocheir japonicus*) and many marine bivalves (Abe *et al.*, 1999; Toyohara *et al.*, 2005). The Japanese mitten crab exhibits increased retention of amino acids, such as l-alanine, in muscle tissue when exposed to increasing salinity (>32 ppt), using free l-alanine as an osmolyte in muscle tissues to regulate osmolarity in an estuarine environment (Abe *et al.*, 1999b). Similarly, the red swamp crayfish (*Procambarus clarkia*) exhibits a 5.4-fold increase in amino acid accumulation in response to increasing salinity in both muscle tissue and the hepatopancreas (Fujimori and Abe, 2002). Likewise, giant Pacific oysters (*Crassostrea gigas*) and the blue mussel *M. galloprovincialis* exhibit increased amino acid transporter expression in the gill epithelia, adductor muscle and kidney in response to both hypoosmotic and hyperosmotic stress (Yamauchi *et al.*, 1992; Toyohara *et al.*, 2005). This could suggest that the utilization of amino acids occurs throughout the body of arthropods and marine invertebrates, not only for protein synthesis but also as an osmolyte and key component in the osmoregulatory capabilities of crustacean species (Abe *et al.*, 1999). Further research is required to conclusively identify the utilization of branchial amino acid uptake in organ tissue and haemolymph of arthropods, as well as arthropods' ability to transport amino acids in varying environmental salinities.

### Environmental conclusions

In order to conserve the diversity and abundance of native species, it is crucial to understand any and all physiological factors that influence species interactions and resultant survival. Throughout this study, we sought to expand our understanding of marine crustacean ion transport processes, and more importantly, gain insight into a potential key factor in the invasive success of the globally dominant *C. maenas*. The invasive green crab is a known euryhaline species with survivable salinity ranging from 4 to 54 ppt (Leignel *et al.*, 2014). This is particularly interesting when compared to the native Pacific crustaceans *Metacarcinus gracilis*, *Metacarcinus magister*, and *Cancer productus*, which all possess a lower ability to regulate internal ions. *M. magister* in particular displays a moderate ability for osmoregulation, surviving prolonged periods in salinity ranging from 12 to 35 ppt (Cleaver, 1949; Sugarman *et al.*, 1983). In contrast, *M. gracilis* and *C. productus* have weak osmoregulatory abilities, unable to withstand extended periods in salinities lower than approximately 18 to 20 ppt and 13 to 16 ppt, respectively (Selby, 1980; Carroll and Winn, 1989; Curtis *et al.*, 2007). With a high thermotolerance (0–35°C), aggressive nature, and extensive thermal tolerance, *C. maenas* leaves no surprise surrounding its ability to thrive in vastly different coastal waters around the world (Leignel *et al.*, 2014). While the invasive green crab displayed the highest capacity for uptake of l-leucine when compared with Canadian native species at high amino acid concentrations, it showed no advantage at environmental levels. However, in the presence of a feeding cloud, or localized area of high amino acid concentrations, the competitive ability of *C. maenas* could be greatly increased. l-leucine was determined to accumulate throughout all tested tissues within the body of *C. maenas*, suggesting widespread utilization. While the exact utilization of these amino acids remains unknown, a deeper understanding of branchial transport could improve clarity regarding success of crustacean species in variable habitats and offer insight into the future spread of *C. maenas*.

## Funding

This work was supported by the Natural Sciences and Engineering Research Council Discovery RGPIN-2020-04153 grant awarded to Dr Blewett.

## Supplementary Data

Supplementary data file contains raw isotope counts, calculations for branchial gill perfusions, and whole body accumulation studies presented in this article.

## Conflict of Interest

We have no competing interests.

## Data Availability Statement

The data underlying this article are available in the Dryad Digital Repository, at https://doi.org/10.5061/dryad.4mw6m90f8

## Ethics

All procedures were approved by the Bamfield Animal Research Ethics Board and the University of Alberta, and were in accordance with the Guidelines for the Canadian Council on Animal Care.

## Author Contributions

T.A.B conceived the design of the study. R.A.G, A.B, A.W, and T.A.B, carried out all experimental procedures. R.A.G carried out all statistical analyses and drafted the manuscript. T.A.B coordinated the study and helped draft the manuscript. R.A.G, A.B, A.W, and T.A.B edited the M.S. All the authors have given their approval for this publication.

## Supplementary Material

Web_Material_coad015
